# Understanding environmental decision making: The association between stages of decision making and decisional conflict

**DOI:** 10.1016/j.joclim.2025.100467

**Published:** 2025-07-24

**Authors:** Letizia Richelli, Eline L.F.M.G. Vissers, Alessandra Gorini, Marijn H.C. Meijers, Eline S. Smit, Thomas Gültzow

**Affiliations:** a University School for Advanced Studies IUSS, Pavia, Italy; b Work and Social Psychology, Faculty of Psychology and Neuroscience, Maastricht University, Maastricht, the Netherlands; c Department of Clinical Sciences and Community Health, University of Milan, Milan, Italy; d Istituti Clinici Maugeri IRCCS, 20138, Milan, Italy; e Department of Communication Science, Amsterdam School of Communication Research/ ASCoR, University of Amsterdam, Amsterdam, the Netherlands; f Department of Theory, Methods & Statistics, Faculty of Psychology, Open University of The Netherlands, Heerlen, the Netherlands

**Keywords:** Pro-environmental behaviours, Stages of decision making, Decisional conflict

## Abstract

Introduction: As research highlights how climate change impacts individual and planetary health, people might consider reducing their ecological footprint by acting pro-environmentally. However, their choice might depend on how far along they are in the process of deciding to act pro-environmentally (i.e., different stages of decision making) and on their experienced difficulty in deciding in favour of the environment and which behaviour to conduct (i.e., decisional conflict).

Methods: To explore in which stage participants find themselves (i.e., not yet thinking about choices, showing interest in doing so, actively considering options, approaching a decision, having already decided but remaining open to reconsideration, or being firmly committed to a decision with little chance of change) and whether their respective stages are correlated with their experienced decisional conflict, an online, cross-sectional questionnaire was distributed among a convenience sample of 418 English-, Dutch-, and Italian-speaking adults to measure participants’ demographics, stages, and extent to which they experience decisional conflict.

Results: Based on regression analyses, we observed that participants who were further along in the stages experienced less decisional conflict.

Conclusion: Therefore, we suggest that interventions (e.g., decision aids) take stages into account to better meet users’ needs and assist people in making environmental decisions.

## Highlights

• People go through different stages when making environmental decisions.
• Individuals experience decisional conflict when making decisions around pro-environmental behaviours.
• Being in later stages of decision making is associated with less decisional conflict.
• Stages of decision making might be taken into account in decision aids designed to support environmental decision making.

## 1 Introduction

### 1.1 Background

Current changes in global weather patterns are attributed to human behaviour and affect people’s physical and mental health [1,2]. Therefore, the effects of climate change are not only an environmental issue but also social and health issues. To address the climate crisis, individuals can reduce their own impact by conducting “pro-environmental behaviours” (PEBs), which aim at reducing the negative effects of consumptive actions on common ecological resources [3]. However, people often find it challenging to engage in PEBs.

#### 1.1.1 Decisional conflict in environmental decision making

One of the factors complicating environmental decision making is that distinct PEBs differ in costs, complexity, and long-term environmental impact [4]. Therefore, deciding to adopt one or more behaviours (over others) might be complex and difficult. This is referred to as *decisional conflict* [5], defined as an “*individual perception of uncertainty about which course of action to take*” [6], p. 61] associated with decisions involving, e.g., uncertainty of outcomes and high-stakes choices with significant potential gains and losses [7]. In environmental decision making, the resources people invest to behave pro-environmentally may be perceived as personal losses [8], yet the outcomes of their actions influence common environmental resources [9]. While individuals face the risks of climate change and its effects, they might struggle to perceive the direct outcomes of their PEBs on the environment [10], making it difficult for people to decide to engage in PEBs.

For over two decades, decisional conflict has been studied mostly in health contexts [11,12], where it has been shown to result in decisional delay [13,14]. However, it has not yet been explored in the context of environmental decision making, where it might similarly play a role in hindering PEB adoption by delaying decisions about whether to act pro-environmentally and how to do so, hence resulting in less PEBs. Studying decisional conflict in the environmental domain is crucial for understanding its impact and effectively addressing these barriers, as doing so is essential for identifying strategies that can support individuals in making informed decisions, thereby informing future intervention studies that use decision aids to enhance the decision-making process [15].

#### 1.1.2 Stages of decision making

Decision aids may be most effective when accounting for people being in different stages of decision making [16]. That is, one person might just begin to think about how to act more pro-environmentally, whereas another may already act pro-environmentally but reconsiders to improve further, and yet another may act pro-environmentally and no longer considers other behaviours. While it is useful to provide more information about available options for those still deliberating, or willing to reconsider their choices, it might be unnecessary when people have already made up their minds. These progressive steps that individuals take to make a decision are referred to as “*stages of decision making*”, hereinafter referred to as “*stages*” [17]. These stages reflect one’s readiness to engage in decision making and range from: if one has not begun to think about the options yet (“precontemplation”); if one has not begun but is interested in getting informed (“interest in contemplation”); if one is considering the option in a specific moment (“contemplation”); if one is close to selecting an option (“close to selection”); if one has already made a decision but is willing to reconsider (“decision, with room for reconsideration”); and if one has already made a decision and is unlikely to change (“decision, with unlikely reconsideration”). Right now, stages are not routinely assessed in decision aid studies nor in studies focusing on decisional conflict [16]. Therefore, we fill a crucial gap in the literature by investigating how stages of decision making and experienced decision conflict are related in environmental decision making.

We posit that the extent to which people experience decisional conflict is linked to the stage they find themselves in. For example, the most conflict could be experienced at the central stages (due to active deliberation processes taking place), while minimal or no conflict may be experienced in the early or final stages when an individual either has not begun to think about the choices yet or has already decided (as both require relatively little cognitive action) - although the relationship also could be linear [16]. In sum, we expect people’s stage to be associated with the extent to which they experience decisional conflict.

Since there are no examples in the literature that apply decisional conflict and stages of decision making in the environmental context, this study aims to connect these two important constructs within this context. Therefore, the research questions (RQs) that this project aims to answer are the following:

**RQ1**: In which decisional stage do individuals find themselves when it comes to deciding to conduct, or not, pro-environmental behaviours?
**RQ2**: Is an individual’s stage of decision making in relation to PEBs related to their experienced environmental decisional conflict?

## 2 Methods

### 2.1 Research design

The present study was part of a bigger, joint project that was registered on the Open Science Framework, where the dataset and the syntax of the analysis can be found [18]. A cross-sectional, explorative, quantitative study using an anonymous questionnaire was distributed and shared on university platforms and social media and hosted on Qualtrics (*Appendix A*). To reach a bigger sample, i.e., to also include participants that lacked the necessary English skills, a convenience sample of English-, Dutch-, and Italian-speaking adults was recruited. Those three languages were chosen as they are spoken by the two lead researchers. We translated back and forth, as indicated by Tsang et al. [19]. The questionnaire fulfilled the criteria listed by Eysenbach [20], i.e., the Checklist for Reporting Results of Internet E-Surveys (*Appendix B*). Due to the observational nature of the study, variables were not manipulated.

### 2.2 Participants

Before starting the data collection, an a priori power analysis was run via G*Power to estimate a sufficient sample (*Appendix C*). Unlike the outcome of the power calculation (*N* = 185), a larger number of people was reached (*N* = 586) to minimize the effect of attrition. Ultimately, 520 Participants were included as they (1) indicated they were 18 years old or older, (2) passed a CAPTCHA test in Qualtrics, (3) agreed to the terms and conditions of the informed consent form, and (4) answered the control questions correctly. Of those, 418 participants were included in the main analysis as (5) they completed all the scales, and were compared to 75 participants who did not complete the questionnaire but answered all the demographic items. Details are provided in *Appendix D*. However, some of the 418 participants indicated that they preferred not to share certain demographic details; these responses were treated as missing data, and those participants were excluded from the regression analyses.

### 2.3 Measurements

Measures of participants’ demographics (i.e., age, gender, income, education, and language), decisional conflict, and stages of decision making related to PEBs were collected. Measurement details are provided in *Appendix E*.

### 2.4 Procedure

This research was approved by the Ethics Review Committee Psychology and Neuroscience (ERCPN) at Maastricht University (*Appendix F*). We did not pilot-test the study, but we delivered a first draft of the completed and translated questionnaire to two English-speaking, two Italian-speaking and two Dutch-speaking people. By doing this, we collected feedback and comments on what and how to improve our items.

In accordance with ethical principles of the American Psychological Association [21], participants read the information letter (*Appendix G*) and gave informed consent (*Appendix H*). Both were provided online before the questionnaire. An online debriefing (*Appendix I*) was given at the end of the questionnaire (*Appendix J*). We did not foresee any negative consequences for participants, and they did not receive any monetary compensation, but undergraduates could obtain 0.5 credits upon completion. The recruitment started in May 2022, and ended in June 2022. Collected data were fully anonymized and treated confidentially.

### 2.5 Analysis

Analyses were run according to the registration, in SPSS Statistics (Version 27.0). Scale diagnosis (e.g., principal component analysis) was performed for the decisional conflict scale, which consisted of multiple items. Based on this, the extent to which people experience decisional conflict was recoded to reflect scores ranging from 0 (no decisional conflict) to 100 (extremely high decisional conflict). To investigate in which stages participants find themselves regarding environmental decision-making (**RQ1**) and whether this is related to decisional conflict (**RQ2**), descriptive statistics were run for decisional conflict and stages. These data were first plotted separately, then together, to investigate the nature of their relationship. Because their scatterplot did not show a clear (but near) linear relationship, a logistic regression was run, followed by a linear regression as sensitivity analysis. Decisional conflict was categorized into low and high based on the commonly used cut-off value of 37.5 for the logistic regression [22]. In the first block, demographics (i.e., age, gender, income, education, and language) were entered with decisional conflict as dependent variable. In the second block, stages were added to test whether including stages improved the model, i.e., explaining more variance in decisional conflict. Then, to explore language differences within the sample, analyses were conducted once more for each language group separately. Moreover, to see whether some participants (in terms of demographics) were more likely to drop out than others, we compared dropouts with those who fully completed the questionnaire. For language and gender, they were compared by means of Chi-square tests of homogeneity (Rx2). For age, income, and education, the assumptions of the Chi-square test were violated, therefore a Fisher’s exact test was conducted.

## 3 Results

### 3.1 Scale diagnosis

Scale diagnosis results are provided in Appendix K.

### 3.2 RQ1: in which decisional stage do individuals find themselves when it comes to deciding to conduct, or not, pro-environmental behaviours?

**Fig. 1:**
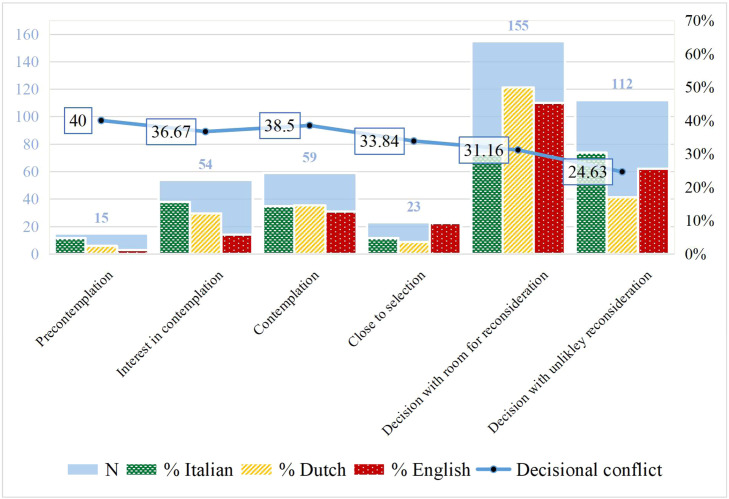
Overlay of Stages of Decision Making histogram x Mean of Decisional Conflict line with Language Groups percentage distribution, and overall sample distribution in the background. Note. Precontemplation: haven’t begun to think about the choices; Interest in contemplation: haven’t begun to think about the choices, but are interested in doing so; Contemplation: are considering the options now; Close to selection: are close to selecting an option; Decided, with room for reconsideration: have already made a decision, but are still willing to reconsider; Decided, with unlikely reconsideration: have already made a decision and are unlikely to change their mind.

More than half of participants reported being in the later stages (i.e., 37.1 % had already made a decision but were still willing to reconsider; and 26.8 % had already made a decision and were unlikely to change their mind). A smaller percentage reported to be in earlier stages (i.e., 3.6 % had not begun to think about the choices; and 12.9 % had not begun to think about the choices, but were interested in doing so) or the central stages of active deliberation (i.e., 14.1 % were considering the options; and 5.5 % were close to selecting an option). Participants' decisional stages and conflicts are provided in Table 1, and a visual representation of participants’ distribution across languages is shown in Fig. 1.

**Table 1:** Descriptives of Stages of decision making and Decisional conflict (*N* = 418).

Stages of decision Making		Experienced Decisional Conflict		
	N	Mean	Std. Deviation	Std. Error
1. Precontemplation: haven't begun to think about the choices;	15	40.00	15.42	3.98
2. Interest in contemplation: haven't begun to think about the choices, but are interested in doing so;	54	36.67	16.88	2.30
3. Contemplation: are considering the options now;	59	38.50	15.30	1.99
4. Close to selection: are close to selecting an option;	23	33.84	11.69	2.44
5. Decision, with room for reconsideration: have already made a decision, but are still willing to reconsider;	155	31.16	14.14	1.14
6. Decision, with unlikely reconsideration: have already made a decision and are unlikely to change your mind.	112	24.63	12.21	1.15

Note. Stages: *M* = 4.40; Mdn= 5.00; SD = 1.51; Skew = −0.71; Kurt = −0.77. Decisional conflict: *M* = 31.62; Mdn= 30.00; SD = 14.95; Skew = 0.23; Kurt = −0.08.

### 3.3 RQ2: is an individual’s stage of decision making in relation to PEBs related to their experienced environmental decisional conflict?

Descriptives showed that decisional conflict scores ranged from 0 to 81.67. Participants in the earliest stage (i.e., precontemplation) showed the highest mean scores of decisional conflict and participants in the latest stage (i.e., decision, with unlikely reconsideration) showed the lowest mean scores of decisional conflict. Based on the cut-off value of high and low decisional conflict, people in later stages (i.e., close to selection; decision, with room for reconsideration; decision, with unlikely reconsideration) showed low decisional conflict, and generally lower decisional conflict than people in the earlier stages (i.e., precontemplation; interest in contemplation; contemplation). However, values did not decrease gradually, as participants who were “considering the options” at that time reported a similar decisional conflict as the participants in the previous stage, making the relationship between stages and decisional conflict (slightly) non-linear.

The logistic regression showed that stages of decision making were significantly associated with lower decisional conflict scores (*B* = −0.47, 95 % CI 0.53 to 0.75, *p* < .001), i.e., being in later stages, compared to earlier stages, was associated with a lower level of decisional conflict. With the addition of stages, the model of demographics on decisional conflict changed from χ2(6) = 31.66, p < 0.001, to χ2(7) = 60.62, *p* < .001, and explained first 11.8 %, then 21.8 % (Nagelkerke R2) of the variance in decisional conflict after stages were entered. Only two predictors were statistically significant in the first model: language (speaking Italian *B* = −1.38, 95 % CI 0.14 to 0.45, *p* < .001; speaking English *B* = −0.84, 95 % CI 0.22 to 0.87, *p* = .025) and age (*B* = −0.22, 95 % CI 0.66 to 0.98, *p* = .032), i.e., speaking Italian or English, compared to Dutch, and being older relative to younger, were associated with a lower level of decisional conflict. Once the stages were entered only language and stages remained significant (speaking Italian *B* = −1.50, 95 % CI 0.12 to 0.41, *p* < .001), i.e., speaking Italian (compared to Dutch) was associated with a lower level of decisional conflict. The area under the Receiver Operating Characteristic (ROC) curve was 0.74, 95 % CI 0.68 to 0.79, which is an acceptable level of discrimination according to Hosmer et al. [23]. Details are reported in Table 2.

**Table 2:** Logistic regression predicting decisional conflict from age, gender, education, income, language and stages of decision making (*N* = 353).

Variable	B	S.E.	Exp(B)	95 % C.I. for EXP(B)	
				Lower	Upper
**Model 1**					
Education	−0.28	0.24	0.75	0.47	1.20
Language					
Italian^1^	−1.38**	0.29	0.25	0.14	0.45
English^1^	−0.84*	0.35	0.43	0.22	0.87
Age	−0.22*	0.10	0.80	0.66	0.98
Income	0.02	0.05	1.02	0.93	1.13
Gender					
Women and non-binary^2^	−0.08	0.26	0.92	0.56	1.52
Constant	1.37	0.70	3.95		
**Model 2**					
Education	−0.15	0.25	0.86	0.53	1.41
Language					
Italian^1^	−1.50**	0.31	0.22	0.12	0.41
English^1^	−0.56	0.37	0.57	0.28	1.18
Age	−0.10	0.11	0.90	0.73	1.11
Income	0.02	0.05	1.02	0.92	1.13
Gender					
Women and non-binary^2^	−0.10	0.27	0.91	0.54	1.53
Stages	−0.47**	0.09	0.63	0.53	0.75
Constant	2.83**	0.80	16.97		

Note. *p < .05, **p < .001, ^1^reference: Dutch, ^2^reference: men.

Similarly, the linear regression showed that later stages were significantly associated with lower decisional conflict scores (*B* = −3.44, 95 % CI −4.41 to −2.48, *p* < .001), i.e., being in later stages, compared to earlier stages, was associated with a lower level of decisional conflict. With the addition of stages, the model of demographics on decisional conflict changed from R2 = 0.20, F(6, 346) = 14.36, p < 0.001 [adjusted R2 = 0.19] to R2 = 0.30, F(7, 345) = 21.09, p < 0.001 [adjusted R2 = 0.29]. Only two predictors were statistically significant in the first model: language (speaking Italian *B* = −14.07, 95 % CI −17.71 to −10.42, *p* < .001; speaking English *B* = = −6.89, 95 % CI −11.49 to −2.30, *p* = .003) and age (*B* = −1.98, 95 % CI −3.19 to −0.78, *p* = .001), i.e., speaking Italian or English, compared to Dutch, and being older relative to younger, were associated with a lower level of decisional conflict. Once the stages were entered only language and stages remained significant (speaking Italian *B* = −13.83, 95 % CI −17.24 to 10.42, *p* < .001; speaking English *B*= −4.39, 95 % CI −8.75 to −0.03, *p* < .05), i.e., speaking Italian or English, compared to Dutch, was associated with a lower level of decisional conflict. Details are reported in Table 3.

**Table 3:** Linear regression predicting decisional conflict from age, gender, income, education, language and stages of decision making (*N* = 353).

Variable	B	S.E.	β	95 % C.I. for EXP(B)	
				Lower	Upper
**Model 1**					
Education	−2.36	1.47	−0.08	−5.25	0.54
Language					
Italian^1^	−14.07**	1.85	0.38	−17.71	−10.42
English^1^	−6.89*	2.34	−0.18	−11.49	−2.30
Age	−1.98*	0.61	−0.18	−3.19	−0.78
Income	−0.05	0.32	−0.01	−0.68	0.59
Gender					
Women and non-binary^2^	0.74	1.58	0.02	−2.37	3.86
Constant	37.64**	3.99		29.81	45.48
R2	.20				
F	14.36**				
ΔR2	.20				
ΔF	14.36**				
**Model 2**					
Education	−1.20	1.39	−0.04	−3.93	1.53
Language					
Italian^1^	−13.83**	1.73	0.38	−17.24	−10.42
English^1^	−4.39*	2.22	−0.12	−8.75	−0.03
Age	−1.10	0.59	−0.10	−2.26	0.06
Income	−0.10	0.30	−0.02	−0.70	0.50
Gender					
Women and non-binary^2^	0.49	1.48	0.02	−2.43	3.41
Stages	−3.44**	0.49	−0.33	−4.41	−2.48
Constant	48.16**	4.02		40.25	56.07
R2	.30				
F	21.09**				
ΔR2	.10				
ΔF	49.38**				

Note. *p < .05, **p < .001, ^1^reference: Dutch, ^2^reference: men.

### 3.4 Language comparison

The effect of stages on decisional conflict was overall stable within the different language groups and both logistic as well as linear regressions (except for the English-speaking sample, in the logistic regression). The effect of age on decisional conflict was present in the full sample only and disappeared when stages were added to the regression. Details of the differences are reported in Table 4, while the results per regression are explained in Appendix L.

**Table 4:** Differences in Language groups.

	Logistic regression		Linear regression	
	Model 1	Model 2	Model 1	Model 2
	Demographics model only	Full model with stages	Demographics model only	Full model with stages
Full sample	Language and age were associated with a lower level of decisional conflict.	Only stages and language were associated with a lower level of decisional conflict.	Language and age were associated with a lower level of decisional conflict.	Only stages and language were associated with a lower level of decisional conflict.
Italian	Model was not significant, *p* > .05.	Only stages were associated with a lower level of decisional conflict.	Age was associated with a lower level of decisional conflict, but the model was not significant, *p* > .05.	Only stages were associated with a lower level of decisional conflict.
English	Model was not significant, *p* > .05.	Model was not significant, *p* > .05.	Model was not significant, *p* > .05.	Only stages were associated with a lower level of decisional conflict.
Dutch	Model was not significant, *p* > .05.	Only stages were associated with a lower level of decisional conflict.	Age was associated with a lower level of decisional conflict, but the model was not significant, *p* > .05.	Only stages were associated with a lower level of decisional conflict.

### 3.5 Group comparison

Chi-square test comparison of the participants who dropped out after completing the demographic items and participants who did fill in at least one PEB item did not show significant differences in language or gender, and Fisher's exact test comparison did not show significant differences in age or income. However, the two groups differed significantly on education (p < 0.001). Further multiple Fisher's exact tests (2 × 2), corrected with a separately calculated Bonferroni, revealed significant differences between groups only in the proportion of people with a low level education (χ2(2) = 20.90, p < 0.001) who dropped out from the study after filling the demographic items rather than filling at least one PEB item (n = 13, 18.1 % versus n = 13, 3.1 %), meaning people with a low level of education tended not to complete the questionnaire, relative to people with a higher level of education.

## 4 Discussion

### 4.1 Principal findings

The aim of this study was to investigate which decisional stage individuals find themselves in when it comes to deciding to conduct, or not, PEBs (RQ1), to which extent they experience decisional conflict in this context, and whether their stage is associated with the decisional conflict they experience (RQ2). Regarding RQ1, a large proportion of the sample reported to be in later stages, meaning that not only they believe they are aware of the different pro-environmental options, but also that they already made a decision about these. Regarding RQ2, we found that stages were negatively associated with decisional conflict, meaning that participants in later stages showed lower levels of decisional conflict. Furthermore, stages significantly explained some of the variance of decisional conflict. Together, our findings indicate that, as higher conflict is experienced in earlier and central stages, it is important to support people advancing through stages in their decision to conduct PEBs, as initial conflict might keep them from implementing those behaviours. This provides much needed initial insights on the stages of decision making in the environmental context and the relationship with decisional conflict.

### 4.2 Secondary findings and implications

Although this was the first study to explore stages and decisional conflict in an environmental context, this association has already been investigated in a health context, where these measures are used to help clinicians enhance patients’ involvement and readiness in decision making, adherence to treatment, and clarification of values, to improve informed, shared decision making [5,[24], [25], [26]].

In line with our findings, Gültzow et al. [16] found a linear association between stages and decisional conflict in smokers receiving decision aids for smoking cessation, with smokers in later stages experiencing less decisional conflict. Similarly, Bailey et al. [27] also found that later stages were associated with lower decisional conflict in diabetes mellitus patients requiring treatment intensification. However, Murray et al. [28] reported a different pattern in women deciding on end-of-life care. Unlike our findings, but in line with what we originally expected, they reported that the highest decisional conflict was experienced in central stages (i.e., active deliberation), followed by the later stages (i.e., finished deliberation), and the lowest in earlier stages (i.e., preliminary stages). These differences highlight the complexity of stage-related decisional conflict, particularly as stages are infrequently assessed together with decisional conflict, making it challenging to describe their relationship comprehensively [16].

Regarding the relationships of demographics with stages on decisional conflict, we found that age was associated with lower decisional conflict, but this effect disappeared when stages were added to the model, meaning that it is possible that the association between age and decisional conflict may be confounded. A possible explanation for this is that older individuals might feel (more) certain about their decisions, thus scoring higher on the stages scale, because of the experience they have accumulated in life. Although the effect of aging on decision-making process effectiveness has been extensively investigated for medical reasons [29], it is still unclear if age directly affects decisional conflict levels or does so indirectly via stages. Future research could investigate this in more detail, also regarding the environmental context. The chosen language also had a significant effect on decisional conflict, i.e., speaking Italian and English was associated with less decisional conflict, relative to speaking Dutch, thus reflecting differences existing among language groups. These differences may be due to language group demographics, as these were not fully homogeneous, as reported in Appendix XI, Table 7. Alternatively, these differences might also be due to each variable’s effect size, that can change in magnitude across sub samples. Finally, all these differences might reflect cultural differences. Although the current study was not set-up to test cultural differences, it would be interesting for future research to investigate whether and how cultural differences affect the relationship between stages and decisional context. While doing so, it would be interesting to also compare the Global North with the Global South as the urgency of environmental problems might differ, thus affecting decisional conflict.

In terms of attrition, there were significant differences in the educational level of participants who partially filled out the questionnaire or completed it. Those with a low level of education were less likely to complete the questionnaire, compared to those with a medium or higher education. This, while unfortunate, is in line with previous research [30], and should be a focus of future efforts to improve inclusivity in data collection.

### 4.3 Practical applications

We believe the results in this study are valuable for future research and intervention development that focus on PEBs, e.g., as depicted by Kunreuther and Weber [31]. As stages may be associated with PEB implementation, we believe that measuring stages might be a valuable starting point for decision aids supporting different kinds of PEBs. Therefore, we suggest that decision aid interventions take stages into account when addressing decisional conflict in the environmental context.

Furthermore, it would be good to tailor decision aids approaches to different stages, as individuals’ responses to decision support depend on their respective stage [17]. Although stage-matched designs are not always superior to stage-mismatched ones in promoting health behaviours, e.g., when testing interventions according to stages of change, a similar yet different concept from stages of decision-making investigated here [32,33], it might be relevant to test whether tailoring works better in the environmental context [34].

When considering tailoring to stages of decision making, one could suggest the following: for those who have not begun to think about the choices, decision aids could focus on increasing awareness and motivation; for those who are considering the options, decision aids could provide details about such options and help clarify values; and for those who have already made a decision, decision aids could be provided to ascertain whether their decision are oriented with their values and based on enough information, in addition to support decision implementation and behaviour change [35]. Also, our findings indicate that especially those in early stages benefit from “traditional” decision aids in the first place, as they experience more conflict.

While the decisional conflict scale allowed us to measure uncertainty around deciding between different PEBs, the causes of this uncertainty (e.g., the behaviours’ impact, affordability, and attainability) were not explored. Measuring stages could also help explain the causes of uncertainty (e.g., by exploring the different reasons that are associated with each stage, we might find, for instance, that financial barriers are most frequently experienced by people in earlier stages), but this should be investigated further.

Moreover, demographic factors also seem important in explaining decisional conflict around environmental considerations, but they might not be as predictive of decisional conflict as stages are and generally seem to have less of an (direct) impact overall. Future studies could explore this further.

Finally, the observed relationship in this study reflects people’s decision processes around environmentally friendly behaviours without differentiating between types of PEBs, as this broader focus offers insights into the challenges people face when navigating complex and diverse options for environmentally friendly action. For future research, it might be interesting to investigate the link between stages and conflict for specific PEBs.

### 4.4 Limitations

Self-reported answers in questionnaires do not always reflect the objective reality of one’s behaviour [36]. Nevertheless, because participants did not receive monetary compensation, this bias was considered minimal. Additionally, data were cross-sectional and did not come from an experimental study, so causal inference could not be explored. The lower completion rate among individuals with a lower level of education highlights the accessibility challenges faced by our psychological study across all educational levels. To address this issue, future researchers should consider potential solutions, such as using simplified, low-literacy versions of the scales used in this study, e.g., the scale developed by Linder et al. [37] to measure decisional conflict. The prevalence of educated people completing the survey also likely explains the high proportion we found in later stages of environmental decision making, as research shows that this group reports high knowledge on climate change and what can be done by individuals to address it, i.e., PEBs [38].

## 5 Conclusion

Our research shows that stages and decisional conflict are decision-making constructs that can be adapted from the health context to the environmental context. Because stages are associated with decisional conflict, not only should they be included in the development of decision aids that support people’s pro-environmental decisions, but tailoring decision aids to different stages could also be valuable. In addition, researchers could further investigate how stages can be used to explain decisional conflict causes and how cultural differences affect the stages, decisional conflict, and their relationship around environmental considerations.

## Funding sources

This research did not receive any specific grant from funding agencies in the public, commercial, or not-for-profit sectors. M. H. C. M. was supported by an awarded Dutch Research Council (NWO) grant [VI.Veni.201S.075].

## CRediT authorship contribution statement

**Letizia Richelli:** Writing – original draft, Methodology, Investigation, Formal analysis, Data curation, Conceptualization. **Eline L.F.M.G. Vissers:** Methodology. **Alessandra Gorini:** Supervision. **Marijn H.C. Meijers:** Writing – review & editing, Supervision. **Eline S. Smit:** Writing – review & editing, Supervision. **Thomas Gültzow:** Writing – review & editing, Supervision, Resources, Project administration, Methodology, Conceptualization.

## Declaration of competing interest

The authors declare that they have no known competing financial interests or personal relationships that could have appeared to influence the work reported in this paper.

## Appendix Supplementary materials

Image, application 1
