# Supplementary material for: Understanding environmental decision making: The association between stages of decision making and decisional conflict
Source: J Clim Chang Health. 2025 Jul 24;24:100467. doi: 10.1016/j.joclim.2025.100467 (PMC12851303; doi:10.1016/j.joclim.2025.100467)
Supplement: Supplementary file 1 [file mmc1.docx]

# **Appendix A: Data Management**

**Procedures online recruitment**. We are aware that BOTS can also occur in studies where there is no reward for participation, especially when recruiting via social media. Thus, we made use of tools for filtering data.

**Tools for filtering data/checking for BOTS**. To better recognize potential BOTS, but also to check whether participants were paying attention filling the questionnaire, we used:

- The Captcha option in Qualtrics (“I am not a robot”)
- Control questions

1. This is a control question, please choose answer option 4.
2. This is a control question, please choose answer option 6.

- Additional, multiple choice control questions about the content of the study, for those who selected “Yes” to the question: “Are you a bachelor student and do you want to receive the 0.5 Sona credits for filling the questionnaire?”
  1. This is a control question about the content of the study. What is this study about?
  2. This is a control question. What was investigated in this questionnaire?

**Assessing research data for BOTS**. Additional checking has been done by checking potential irregularities in the data.

Data from the questionnaire will be stored in a safe storage environment whether and for as long as the University of Maastricht requires them.

# **Appendix B. Checklist for Reporting Results of Internet E-Questionnaires (CHERRIES)**

| **Item Category** | **Checklist Item** | **Explanation** | **Explanation related to our study** |
| --- | --- | --- | --- |
| **Design** | Describe questionnaire design | Describe target population, sample frame. Is the sample a convenience sample? (In “open” questionnaires this is most likely.) | Participants were included when they (1) indicated they were 18 years old or older, (2) passed a captcha test in Qualtrics, (3) agreed to the terms and conditions of the informed consent form, and (4) answered the control questions correctly. Additionally, participants were included in the main analysis when (5) they completed at least the first item of the pro-environmental behaviour scale. Therefore, the sample frame consisted of virtually all adults who spoke English, Italian or Dutch and had access to the internet. The sample was a convenience sample in the sense that anyone willing to participate could access the questionnaire. |
| **IRB (Institutional Review Board) approval and informed consent process** | IRB approval | Mention whether the study has been approved by an IRB. | The study obtained ERCPN ethical permission from Maastricht University for a single study within the existing research line of Prof. Ruiter’s “Determinanten van Gedrag” (188_10_2_2018_S104). |
|  | Informed consent | Describe the informed consent process. Where were the participants told the length of time of the questionnaire, which data were stored and where and for how long, who the investigator was, and the purpose of the study? | Both Information Letter and Informed consent were provided online before the questionnaire. Informed consent was attained by asking participants to continue only if they were willing to participate and if they had read and understood the instructions and information provided, that were, the duration and procedure of the study, the privacy statement, and researchers contact details. Participants were told that participation was voluntary and that they had the right to withdraw from the study at any time. |
|  | Data protection | If any personal information was collected or stored, describe what mechanisms were used to protect unauthorized access. | Any personal data that was collected was kept to a minimum and deleted afterwards, so that no personal data was stored. This was particularly relevant for a minority of participants who accessed the survey through the university platform with their university credentials, this collected data was subsequently deleted. |
| **Development and pre-testing** | Development and testing | State how the questionnaire was developed, including whether the usability and technical functionality of the electronic questionnaire had been tested before fielding the questionnaire. | To develop the questionnaire, some new self-reported scales were created for this study, and permission was asked in order to include existing scales and, if and where possible, accurately adapt them to the environmental topic. To test its usability and functionality, we delivered a first Qualtrics draft of the completed questionnaire to two English-speaking, two Italian-speaking and two Dutch-speaking people. By doing this, we collected feedback and comments on what and how to improve our items. |
| **Recruitment process and description of the sample having access to the questionnaire** | Open questionnaire versus closed questionnaire | An "open questionnaire" is a questionnaire open for each visitor of a site, while a closed questionnaire is only open to a sample which the investigator knows (password-protected questionnaire). | The questionnaire was open. |
|  | Contact mode | Indicate whether or not the initial contact with the potential participants was made on the Internet. (Investigators may also send out questionnaires by mail and allow for Web-based data entry.) | Links to recruit participants were distributed through private messages, spread within the network of the researchers, and shared on university platforms (e.g., SONA) and on different social media platforms (e.g., Instagram, LinkedIn, Facebook). |
|  | Advertising the questionnaire | How/where was the questionnaire announced or advertised? Some examples are offline media (newspapers), or online (mailing lists – If yes, which ones?) or banner ads (Where were these banner ads posted and what did they look like?). It is important to know the wording of the announcement as it will heavily influence who chooses to participate. Ideally the questionnaire announcement should be published as an appendix. | Online recruitment took place by making use of unpaid social media posts shared on the team members' SONA, Instagram, LinkedIn, and Facebook accounts. Such social media accounts are still online, and so are all recruitment posts. See for example LinkedIn: <https://www.linkedin.com/posts/thomasgultzow_hi-everyone-letizia-and-i-are-working-activity-6939980991214034944-AC2R?utm_source=share&utm_medium=member_desktop> |
| **Questionnaire administration** | Web/E-mail | State the type of e-questionnaire (e.g., one posted on a Web site, or one sent out through e-mail). If it is an e-mail questionnaire, were the responses entered manually into a database, or was there an automatic method for capturing responses? | The questionnaire was created and embedded into Qualtrics. |
|  | Context | Describe the Web site (for mailing list/newsgroup) in which the questionnaire was posted. What is the Web site about, who is visiting it, what are visitors normally looking for? Discuss to what degree the content of the Web site could pre-select the sample or influence the results. | See the checklist item *Advertising the questionnaire*. |
|  | Mandatory/voluntary | Was it a mandatory questionnaire to be filled in by every visitor who wanted to enter the Web site, or was it a voluntary questionnaire? | The link for the online questionnaire was voluntary for participants to access. |
|  | Incentives | Were any incentives offered (e.g., monetary, prizes, or non-monetary incentives such as an offer to provide the questionnaire results)? | Participants did not receive any monetary compensation. Students at Maastricht University could receive 0.5 SONA credit if they indicated in the questionnaire their SONA identification number, completed the questionnaire, and answered correctly all the control questions. |
|  | Time/Date | In what timeframe were the data collected? | Data was collected between May 2022 and June 2022. |
|  | Randomization of items or questionnaires | To prevent biases items can be randomized or alternated. | Items were neither randomized nor alternated. |
|  | Adaptive questioning | Use adaptive questioning (certain items, or only conditionally displayed based on responses to other items) to reduce number and complexity of the questions. | Some items were automatically adapted depending on the respective answers. Specifically, if participants did not give online consent to the study, they were redirected to the end of the questionnaire and no data was collected. Moreover, if they were students at Maastricht University and they needed 0.5 SONA credit for completing the questionnaire, they were displayed one item asking for their SONA identification number and two more control questions. |
|  | Number of Items | What was the number of questionnaire items per page? The number of items is an important factor for the completion rate. | The number of items per page varied per page. Demographic measures were assessed with 4 items, Stages of Decision Making with 1 item, Decisional conflict with 16 items. |
|  | Number of screens (pages) | Over how many pages was the questionnaire distributed? The number of items is an important factor for the completion rate. | The questionnaire resulted in 11 online pages of different lengths and items (e.g., one page only for the Captcha item). The questionnaire resulted in 23 pages of different lengths and items in the pdf version, comprehensive of Information Letter, Informed consent, and Debriefing. |
|  | Completeness check | It is technically possible to do consistency or completeness checks before the questionnaire is submitted. Was this done, and if “yes”, how (usually JAVAScript)? An alternative is to check for completeness after the questionnaire has been submitted (and highlight mandatory items). If this has been done, it should be reported. All items should provide a non-response option such as “not applicable” or “rather not say”, and selection of one response option should be enforced. | Because we used forced-choice questions, all submitted versions of the questionnaire were considered filled. Demographics items included the option “Prefer not to say” that was re-coded as Missing data. Data from participants who did not complete the questionnaire but answered at least demographics items were included for the group comparison analysis. |
|  | Review step | State whether respondents were able to review and change their answers (e.g., through a Back button or a Review step which displays a summary of the responses and asks the respondents if they are correct). | Participants could not change their answers once they moved to the next page. |
| **Response rates** | Unique site visitor | If you provide view rates or participation rates, you need to define how you determined a unique visitor. There are different techniques available, based on IP addresses or cookies or both. | Participation and view rates were not collected. To determine a unique visitor, Qualtrics registered IP addresses and did not allow participants to access the questionnaire twice once it was completed. |
|  | View rate (Ratio of unique questionnaire visitors/unique site visitors) | Requires counting unique visitors to the first page of the questionnaire, divided by the number of unique site visitors (not page views!). It is not unusual to have view rates of less than 0.1 % if the questionnaire is voluntary. | Information not available, see *Unique site visitor*. |
|  | Participation rate (Ratio of unique visitors who agreed to participate/unique first questionnaire page visitors) | Count the unique number of people who filled in the first questionnaire page (or agreed to participate, for example by checking a checkbox), divided by visitors who visit the first page of the questionnaire (or the informed consents page, if present). This can also be called “recruitment” rate. | Information not available, see *Unique site visitor*. |
|  | Completion rate (Ratio of users who finished the questionnaire/users who agreed to participate) | The number of people submitting the last questionnaire page, divided by the number of people who agreed to participate (or submitted the first questionnaire page). This is only relevant if there is a separate “informed consent” page or if the questionnaire goes over several pages. This is a measure for attrition. Note that “completion” can involve leaving questionnaire items blank. This is not a measure for how completely questionnaires were filled in. (If you need a measure for this, use the word “completeness rate”.) | Depending on the measurement points: participants who completed the demographics items: 75/520 (14.42%); participants who completed the first item of the last scale: 418/520 (80,38%). The total number of 520 does not include those participants who did not meet the inclusion criteria (e.g., younger than 18 years old). |
| **Preventing multiple entries from the same individual** | Cookies used | Indicate whether cookies were used to assign a unique user identifier to each client computer. | Not applicable |
|  | IP check | Indicate whether the IP address of the client computer was used to identify potential duplicate entries from the same user. If so, mention the period of time for which no two entries from the same IP address were allowed (e.g., 24 hours). Were duplicate entries avoided by preventing users with the same IP address access to the questionnaire twice; or were duplicate database entries having the same IP address within a given period of time eliminated before analysis? If the latter, which entries were kept for analysis (e.g., the first entry or the most recent)? | The IP address of the participant device was used by Qualtrics to identify potential duplicate entries from the same user. There was no period of time for which two or more entries from the same IP address were not allowed, this means, the participant could start and finish the questionnaire in two or more accesses, but could not start again or access the questionnaire twice once completed. Once we closed the questionnaire on Qualtrics and started analysing the data (i.e., one month after the recruitment), participants could not access the questionnaire anymore. |
|  | Log file analysis | Indicate whether other techniques to analyse the log file for identification of multiple entries were used. | Not applicable. |
|  | Registration | In “closed” (non-open) questionnaires, users need to login first and it is easier to prevent duplicate entries from the same user. Describe how this was done. | Not applicable. |
| **Analysis** | Handling of incomplete questionnaires | Were only completed questionnaires analyzed? Were questionnaires which terminated early (where, for example, users did not go through all questionnaire pages) also analyzed? | Participants who filled in at least one item of the pro-environmental behaviours scale (inclusion criterion 5) were considered fort the main analyses. Participants that did not complete at least the first item of the pro-environmental behaviours scale were compared by means of Chi-square tests to those who did to see if they differed in regards to demographic variables. |
|  | Questionnaires submitted with an atypical timestamp | Some investigators may measure the time people needed to fill in a questionnaire and exclude questionnaires that were submitted too soon. Specify the timeframe that was used as a cut-off point and describe how this point was determined. | Not applicable. |
|  | Statistical correction | Indicate whether any methods such as weighting of items or propensity scores have been used to adjust for the non-representative sample; if so, please describe the methods. | Not applicable. |

# **Appendix C: G*Power Calculation**

Sample population was estimated with G*Power (Version 3.1.9.7). The number of subjects was calculated for every RQ of the joint project, and the one which required the most participants was considered for the recruitment. For that RQ, a linear multiple regression (fixed model, R^2^ increase) was selected from the family of F tests on G*Power. Because an effect size of decisional conflict on PEBs had not been described in the literature, a small to medium effect size f^2^ (r = 0.085) was assumed. Next, we aimed for a power of 0.95 and an alpha error probability of 0.05. Two tested predictors (i.e., the independent variables of interest) and 16 total predictors (i.e., the former ones together with all the covariates) were considered for the power calculation. The total predictors were gender turned into two dummy variables (i.e., gender turned into two dummy variables (1: woman and non-woman, 2: non-binary and binary, using men as a reference category), age, education, income, one variable for the preference for intuition and deliberation, decisional conflict, one variable for perceived norms, one variable for attitudes, perceived behavioural control, actual control, intention, response efficacy, motivation, and language turned into two dummy variables (1: English- and non-English speakers, 2: Italian- and non-Italian speakers, using Dutch as a reference category). The outcome of the power calculation resulted in a required sample size of 185 participants. For visual representation see below Figure A.1.

**Figure A.1**

*Screenshot of a G*Power calculation to estimate the required sample size.*


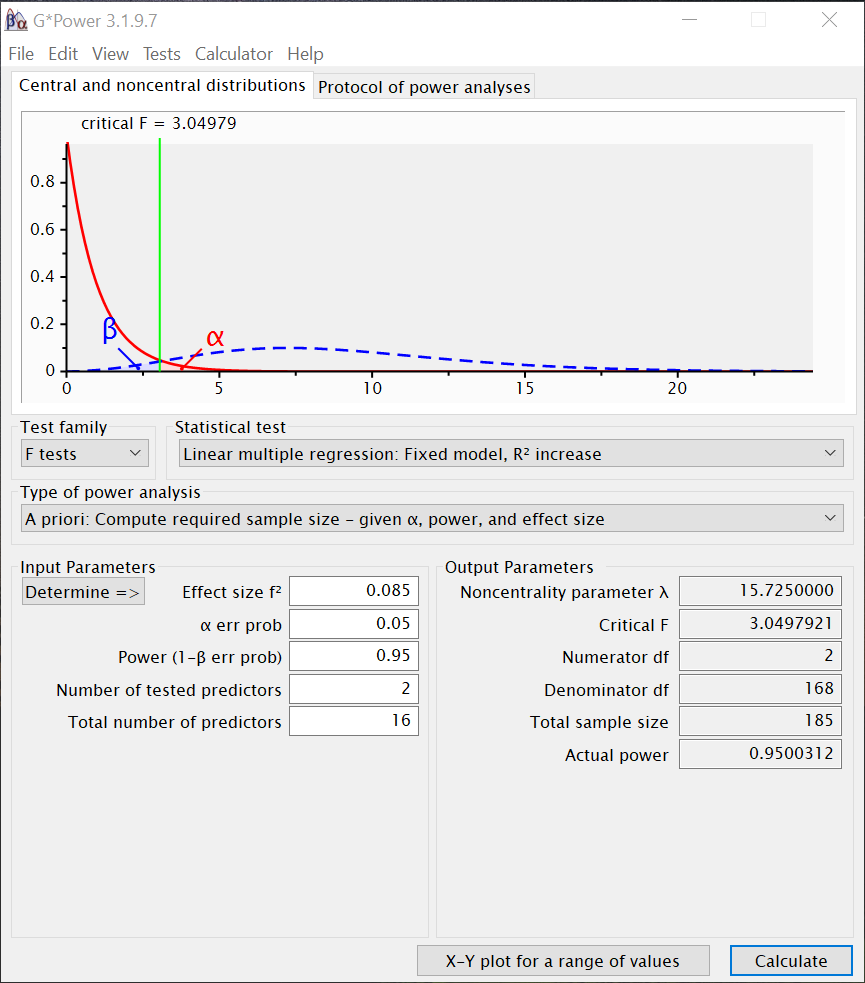


# **Appendix D: Sample results**

A total of 586 participants was initially recruited. Then, 11 were excluded because they were not 18 years old, 37 were excluded because they did not agree to the terms and conditions of the informed consent form, and 18 were excluded because they did not answer the control questions correctly. Finally, the remaining 520 were divided in two groups: 418 participants who filled in all the scales and 75 who did not complete the questionnaire but answered all the demographic items (27 participants were excluded as they did not answer all the demographics). The first was considered for the main analyses, while the second was included in the attrition analysis. Characteristics of both groups are provided in Table D.1.

**Table D.1**

*Frequency table of participants (N=520).*

|  | Entire sample  (n =520) | Participants who filled in at least 1 item of the PEB scale  (n =418) | Participants who filled in at least the demographic items  (n =75) |
| --- | --- | --- | --- |
| **Language, n (%)**  Italian  English  Dutch | **520 (100.0)**  314 (60.4)  106 (20.4)  100 (19.2) | **418 (100.0)**  250 (59.8)  86 (20.6)  82 (19.6) | **75 (100.0)**  49 (65.3)  11 (14.7)  15 (20.0) |
| **Gender, n (%)**  Men  Women  Non-binary  Missing | **501 (96.3)**  169 (32.5)  325 (62.5)  4 (0.8)  22 (4.2) | **415 (99.3)**  142 (34.0)  271 (64.8)  2 (0.5)  3 (0.7) | **75 (100.0)**  24 (32.0)  49 (65.3)  2 (2.7)  0 |
| **Age, n (%)**  18-30  31-40  41-50  51-60  61-70  71-80  >80  Missing | **501 (96.3)**  255 (49.0)  57 (11.0)  69 (13.3)  97 (18.7)  17 (3.3)  6 (1.2)  0  19 (3.7) | **418 (100.0)**  209 (50.0)  47 (11.2)  61 (14.6)  81 (19.4)  15 (3.6)  5 (1.2)  0  0 | **75 (100.0)**  41 (54.7)  10 (13.3)  6 (8.0)  15 (20.0)  2 (2.7)  1 (1.3)  0  0 |
| **Income, n (%)**  < 150€  151€ – 300€  301€ – 500€  501€ – 1000€  1001€ – 1500€  1501€ – 2000€  2001€ – 2500€  2501€ – 3000€  3001€ – 5000€  5001€ – 10.000€  >10.000€  Missing | **430 (82.7)**  27 (5.2)  20 (3.8)  19 (3.7)  55 (10.6)  81 (15.6)  63 (12.1)  43 (8.3)  37 (7.1)  52 (10.0)  22 (4.2)  11 (2.1)  90 (17.3) | **359 (85.9)**  22 (5.3)  15 (3.6)  15 (3.6)  46 (11.0)  58 (13.9)  58 (13.9)  37 (8.9)  30 (7.2)  48 (11.5)  22 (5.3)  8 (1.9)  59 (14.1) | **66 (88.0)**  5 (6.7)  5 (6.7)  4 (5.3)  8 (10.7)  19 (25.3)  5 (6.7)  6 (8.0)  7 (9.3)  4 (5.3)  0  3 (4.0)  9 (12.0) |
| **Education, n (%)**  Low  Medium  High  Missing | **494 (95.0)**  27 (5.2)  181 (34.8)  286 (55.0)  26 (5.1) | **414 (99.0)**  13 (3.1)  159 (38.0)  242 (57.9)  4 (1.0) | **72 (96.0)**  13 (17.3)  19 (25.3)  40 (53.3)  3 (4.0) |

# **Appendix E: Measurements**

| **Measurements** | **Answer categories** | **Data recoding^[[1]](#footnote-1)^ and other informations** |
| --- | --- | --- |
| ***Demographic information*** | | |
| **Gender**  “Which gender do you identify with?” | 1 = Man  2 = Woman  3 = Non-binary  4 = Prefer not to say | Because there were few non-binary cases, and in order not to exclude them from the analyses, this variable was recoded into a dummy (0= Others, 1= Women and non-binary) with Men as reference category. The option “Prefer not to say” was recoded as missing data. |
| **Age**  “What is your age?” | 1 = <18  2 = 18-30  3 = 31-40  4 = 41-50  5 = 51-60  6 = 61-70  7 = 71-80  8 = >80  9 = Prefer not to say | The first category started with “less than 18” years old to identify any participants who filled out the questionnaire regardless of the first inclusion criteria. Data coming from these subjects were not included in the analysis. The option “Prefer not to say” was recoded as missing data. |
| **Educational level**  “What is the highest degree or level of education you have completed?” | 1 = Early childhood education  2 = Primary education  3 = Lower secondary education  4 = Upper secondary education  5 = Post-secondary non-tertiary education  6 = Short-cycle tertiary education  7 = Bachelor's or equivalent level  8 = Master's or equivalent level  9 = Doctoral or equivalent level  10 = Prefer not to say | The variable was recoded into low, medium, and high categories (1= Low, 2= Medium; 3= High)  based on the categorization of the standardized levels of the International Standard Classification of Education [1] used for levels of education in Europe, as well as the Central Bureau of Statistics of the Netherlands [2], and the Italian Ministry of Education, University and Research [3]. In the questionnaire, education item options were presented differently across translations and were not completely equivalent. This was due to categories being chosen to reflect the school system that, for instance, a person who lives in Italy, rather than in the Netherlands, experienced. We assumed Italian-speaking people to refer to the Italian school systems since we recruited them within a network of Italian-speaking people who lived in Italy. The same reasoning applied to Dutch-speaking participants. For English-speaking participants, categories referred to an international classification rather than a specific school system. To obtain a common classification for the analysis, Education was recoded in a variable with three levels. We recoded early, primary, and lower secondary education as a low educational attainment; upper secondary education and a post-secondary non-tertiary education as a medium educational attainment and short-cycle tertiary education, bachelor, master and doctoral programmes as high educational attainment, similarly to Gültzow et al. [4]. The option “Prefer not to say” was recoded as missing data. |
| **Monthly household income**  “What is your net monthly household income? Please refer to the income you receive after subtracting your taxes. If you have a partner or family that you live with, refer to the combined income” | 1 = Less than 150€  2 = Between 151€ and 300€  3 = Between 301€ and 500€  4 = Between 501€ and 1000€  5 = Between 1001€ and 1500€  6 = Between 1501€ and 2000€  7 = Between 2001€ and 2500€  8 = Between 2501€ and 3000€  9 = Between 3001€ and 5000€  10 = Between 5001€ and 10.000€  11= More than 10.000€  12 = Prefer not to say | The scale was adapted from Sproesser et al. [5]. The option “Prefer not to say” was recoded as missing data. |
| ***Stages of Decision Making***  “People may be willing to do something for the environment, meaning consciously choosing to minimise or avoid the negative impact of their actions on the environment. At this time, would you say you:” | 1 = haven't begun to think about the choices;  2 = haven't begun to think about the choices, but are interested in doing so;  3 = are considering the options now;  4 = are close to selecting an option;  5 = have already made a decision, but are still willing to reconsider;  6 = have already made a decision and are unlikely to change your mind. | The traditional 1-item Stages of Decision Making Scale [6], originally designed for clinical practice, was applied to environmental decision making and permission to alter the scale (specifically, the item which was originally designed for medical situation, e.g. “People who will be having heart surgery may be asked to consider self-donating blood before surgery or receiving only volunteer blood from healthy volunteers. At this time, would you say you”) was granted by the Ottawa Hospital Research Institute as the copyright holder, whose suggestion was to avoid using the terms “pro-environmental behaviours” when adapting the scales, preferring alternatives such as “doing something for the environment”. This was suggested because, although the concept is largely spread in the literature, the term “pro-” could represent a bias in priming certain answers rather than others, thus impacting the extent of actions people report to conduct. For the same reason, no example of PEBs was mentioned, in order not to prime people’s answers. |
| ***Decisional conflict***  People can decide to do something for the environment, in a range of behavioral options that go from “*considering the environment in everything they do*” to “*not considering it at all*”.  Considering the option you prefer, please evaluate the following statements.   1. I know which options are available to me. 2. I know the advantages of each option. 3. I know the disadvantages of each option. 4. I am clear about which advantages matter most to me. 5. I am clear about which disadvantages matter most to me. 6. I am clear about which is more important to me (the advantages or the disadvantages). 7. I have enough support from others to make a choice. 8. I am choosing without pressure from others. 9. I have enough advice to make a choice. 10. I am clear about the best choice for me. 11. I feel sure about what to choose. 12. This decision is easy for me to make. 13. I feel I have made an informed choice. 14. My decision shows what is important to me. 15. I expect to stick with my decision. 16. I am satisfied with my decision. | 1 = Strongly Agree  2 = Agree  3 = Neither Agree Nor Disagree  4 = Disagree  5 = Strongly Disagree | The traditional 16-items version of the Decisional Conflict scale [7], originally designed for clinical practice, was adapted to the environmental context, after consultation with experts, due to the non-health focus of this study. Permission to alter the scale (specifically, the introduction to items, which was originally: “A. Which [insert treatment/screening] option do you prefer? Please check one. [Option 1; Option 2; Option 3; Unsure]. B. Considering the option you prefer, please answer the following questions” and items “2. I know the benefits of each option; 3. I know the risks and side effects of each option; 4. I am clear about which benefits matter most to me; 5. I am clear about which risks and side effects matter most; 6. I am clear about which is more important to me (the benefits or the risks and side effects”) was granted by the Ottawa Hospital Research Institute as the copyright holder. Validation of the scale [8] indicated that test-retest reliability coefficient was 0.81, and internal consistency coefficients exceed 0.78. Conditional on the scale diagnosis (see Appendix X), one of 16 items was excluded because it did not show a correlation coefficient greater than \|0.3\|. Decisional Conflict Scale was scored from 0-100 by summing the scores from the 15 items, dividing them by 15 and then multiplying them by 25, as indicated by O’Connor [7]. Correspondent scores range from 0 [no decisional conflict] to 100 [extremely high decisional conflict]. |
| ***Additional measurements***  Among other scales used in the partner project and described in the registration provided on Open Science Framework (<https://osf.io/mgvjb/>), a self-reported PEBs measure was included at the end of the questionnaire that consisted of the most common and impactful behaviours from scales in literature [9]. Answers to this scale were used to create a filter variable and divide two groups of participants: those who completed at least the first item of the PEB scale, hence, most of the survey, and those who did not. Subsequently, a demographic comparison of these two groups was done. | | |

1. UNESCO Institute for Statistics. (2012). International Standard Classification of Education ISCED 2011. <http://uis.unesco.org/sites/default/files/documents/international-standard-classification-of-education-isced-2011-en.pdf>
2. Centraal Bureau voor de Statistiek. Opleidingsniveau [Internet]. [cited 2022 June]. Available from: <https://www.cbs.nl/nl-nl/nieuws/2019/33/verschil-levensverwachting-hoog-en-laagopgeleid-groeit/opleidingsniveau>
3. MIUR Ministero dell’Istruzione. (2018). Sistema educativo di istruzione e di formazione. <https://www.miur.gov.it/web/guest/sistema-educativo-di-istruzione-e-formazione>
4. Gültzow T, Smit ES, Hudales R, Dirksen CD, Hoving C. Smoker profiles and their influence on smokers' intention to use a digital decision aid aimed at the uptake of evidence-based smoking cessation tools: An explorative study. Digit Health. 2020 Dec 29;6:2055207620980241. doi: 10.1177/2055207620980241. PMID: 33473322; PMCID: PMC7783882.
5. Sproesser, G., Aulbach, M., Gültzow, T., & König, L. M. (2022, January 12). Do nutrition knowledge, food preferences, and habit strength moderate the association between Preference for Intuition and Deliberation in Eating Decision-making and dietary intake?. <https://doi.org/10.31234/osf.io/s9bta>
6. O’Connor, A. M. (2000). User Manual- Stage of Decision Making [document on the Internet]. Ottawa: Ottawa Hospital Research Institute, 3 p. <http://decisionaid.ohri.ca/docs/develop/User_Manuals/UM_Stage_Decision_Making.pdf>
7. O’Connor, A. M. (2010). User Manual- Decisional Conflict Scale (16 item statement format) [document on the Internet]. Ottawa: Ottawa Hospital Research Institute, 16 p. <http://decisionaid.ohri.ca/docs/develop/User_Manuals/UM_Decisional_Conflict.pdf>
8. O'Connor, A. M. (1995). Validation of a decisional conflict scale. Medical decision-making, 15(1), 25-30.
9. Lange, F., & Dewitte, S. (2019). Measuring pro-environmental behavior: Review and recommendations. *Journal of Environmental Psychology, 63*, 92-100. <https://doi.org/10.1016/j.jenvp.2019.04.009>

# **Appendix F: Ethics Approval**

**Form single study within approved research line**

Submit to [ercpn-fpn@maastrichtuniversity.nl](mailto:ercpn-fpn@maastrichtuniversity.nl)

| **Research line**  ERCPN code:  Title research line:  Name responsible researcher: | **ERCPN- 188_10_02_2018**  **Determinanten van Gedrag**  **Prof. Dr. Rob Ruiter** |
| --- | --- |
| **Project**  Name applicant:  Title research project: | **Thomas Gültzow**  **Understanding decisional conflict around environmental decisions & the influence of stages of decision making** |
| Environmental considerations are becoming increasingly important and there are multiple behavioural options (i.e., different behaviours) that individuals can choose to improve their environmental impact. Considering the uncertainty in this area (e.g., how influential certain behaviours actually are), one might conclude that the decision to choose for one pro-environmental behaviour over the other – or to pursue multiple such behaviours simultaneously – is relatively hard to make for individuals. However, it is currently unknown if individuals do in fact experience this process as being difficult and if and how this influences their environmental behaviour. Also, considerable heterogeneity is expected to exist regarding how far along people are in this process, i.e., different people may find themselves in different stages of decision making. For example, one person might just start to think about how to behave more environmentally friendly. However, another person may already perform certain pro-environmental behaviours, yet, wants to become even more environmentally friendly. This may influence the perceived difficulty of the choice to be made. During this online study we will (1) study to which extent adult (i.e., ≥ 18 years old) people perceive decisional conflict regarding personal environmental decisions, (2) if their experienced decisional conflict is related to environmental behaviour, (3) in which decisional stages participants find themselves, and (4) if their stage of decision making is related to this decisional conflict. In addition, we will explore whether participants' tendency to make decisions either deliberatively or intuitively influences the decisional conflict they experience.  For this research, we use an online questionnaire that will be distributed among a convenience sample of adults. Consent will be provided online, and we do not foresee any negative consequences for the participants. Participants will not receive any monetary compensation for filling in the questionnaire. | |

# **Appendix G: Information Letter**

**Understanding decisional conflict around environmental decisions & the influence of stages of decision making**

**Purpose**

Dear participant,

The purpose of our study is to examine the processes involved with environmental decision making and environmental behaviours.

**Why are we asking you?**

This is a study for all adults (18 years or older). We are interested in the general population, to see how environmental decisions and behaviours work.

**Voluntary participation**

Your participation in this study is completely voluntary. This means you have the right to withdraw from the study at any time, without any negative consequences.

**Procedure**

We will ask you to fill in a questionnaire about your experience with pro-environmental decision making and certain behaviours. This will take approximately 15 minutes.

**Privacy statement**

No personal data will be stored since this questionnaire is completely anonymous. Research data can be published and re-used in other studies, but only in such a way that they cannot be traced back to you. This concerns the following data:

- Age

- Gender

- Household income

- Education

- Environmental decision making

- Psychological factors of environmental decision making

- Environmental behaviours

**Contact details**

If you wish to contact the responsible researcher and contact persons for questions about this study and your rights as a participant, you can use the following contact details:

Eline Vissers (Master student in Health and Social Psychology)

E-mail: [elfmg.vissers@student.maastrichtuniversity.nl](mailto:elfmg.vissers@student.maastrichtuniversity.nl)

Letizia Richelli (Master student in Health and Social Psychology)

E-mail: [l.richelli@student.maastrichtuniversity.nl](mailto:l.richelli@student.maastrichtuniversity.nl)

Dr. Thomas Gültzow (responsible researcher)

E-mail: [thomas.gultzow@maastrichtuniversity.nl](mailto:thomas.gultzow@maastrichtuniversity.nl)

# **Appendix H: Informed Consent**

**Declaration of Consent for participation in the research study:**

**“Understanding decisional conflict around environmental decisions**

**& the influence of stages of decision making”**

**ERCPN- 188_10_02_2018_S104**

I have been informed of the purpose of the study. I have read the information on the previous page about the study and how my data will be used. I have been able to think about my participation in this study, which is completely voluntary. I have the right to withdraw my consent and leave the study at any time without having to give a reason.

I am aware and agree that the anonymous research data can be used for further scientific research.

I have read the above statements and agree to participate in this study:

- Yes
- No

# **Appendix I: Debriefing**

**“Understanding decisional conflict around environmental decisions**

**& the influence of stages of decision making”**

Thank you for participating in our study!

The purpose of this study is to investigate to what extent adults perceive decisional conflict regarding decisions about doing things for the environment and if decisional conflict affects to what extent individuals engage in doing things for the environment. Furthermore, this study wants to explore in which decisional stages individuals find themselves in regarding environmental behaviours, and if these stages of decision making are related to the extent to which people experience decisional conflict. In addition, we wanted to explore whether participants’ tendency to make decisions either deliberately or intuitively influences the decisional conflict they experience.

This study hopes to increase our current knowledge about the link between decision-making processes and environmental behaviours. Hopefully, future studies will increase our understanding of these decisional processes even further so that eventually interventions can be built that can reinforce environmental behaviours within many individuals of our society.

**Contact details**

If you wish to contact the responsible researcher and contact persons for questions about this study and your rights as a participant, you can use the following contact details:

Eline Vissers (Master student in Health and Social Psychology)

E-mail: [elfmg.vissers@student.maastrichtuniversity.nl](mailto:elfmg.vissers@student.maastrichtuniversity.nl)

Letizia Richelli (Master student in Health and Social Psychology)

E-mail: [l.richelli@student.maastrichtuniversity.nl](mailto:l.richelli@student.maastrichtuniversity.nl)

Dr. Thomas Gültzow (responsible researcher)

E-mail: [thomas.gultzow@maastrichtuniversity.nl](mailto:thomas.gultzow@maastrichtuniversity.nl)

# **Appendix J: Questionnaire scales**

**Demographics**

1. What gender do you identify with?
   1. Man
   2. Woman
   3. Non-binary
   4. Prefer not to say
2. What is your age?
   1. <18
   2. 18-30
   3. 31-40
   4. 41-50
   5. 51-60
   6. 61-70
   7. 71-80
   8. >80
   9. Prefer not to say
3. What is the highest degree or level of education you have completed?
   1. Early childhood education

*Typically, this education level is offered from ages 0 to 5 years.*

- 1. Primary education

*Typically, this education level starts between ages 5 and 7 years and lasts 6 years, until age 10 to 12.*

- 1. Lower secondary education

*Typically, this education level starts between ages 10 and 13 and represents the first 3 years of secondary education.*

- 1. Upper secondary education

*Typically, this education level is a continuation of the lower secondary education level and starts between ages 14 and 16.*

- 1. Post-secondary non-tertiary education

*Typically, this education level refers to vocational programs that are designed to prepare for direct labour market entry, for example, technician diploma, primary professional education etc.*

- 1. Short-cycle tertiary education

*Typically, this education level refers to practically-based, occupationally-specific programs that can also give credit to transfer to Bachelor's or Master's programs. An example of a short-cycle tertiary education program is (higher) technical education, community college education, technician or advanced /higher vocational training, associate degree etc.*

- 1. Bachelor's or equivalent level

*Typically, this education level refers to programs that provide students with intermediate academic and/or professional knowledge, skills, and competencies and are theoretically- based, for example, Bachelor's degree in Psychology.*

- 1. Master's or equivalent level

*Typically, programs at this educational level also require theoretically- and/or professionally-based content and may involve a substantial research component like a research project or thesis that is more advanced than those of the Bachelor's level but do not lead to the award of a doctoral qualification, for example, Master's degree in Health and Social Psychology.*

- 1. Doctoral or equivalent level

*Typically, programs of this educational level are only offered by research-oriented tertiary educational institutions such as university. They are aimed to lead to an advanced research qualification and usually conclude with the submission and defense of a thesis, dissertation, or equivalent work of publishable quality.*

- 1. Prefer not to say

1. What is your net monthly household income?
   *Please refer to the income you receive after subtracting your taxes. If you have a partner or family that you live with, refer to the combined income.*
   1. Less than 150€
   2. Between 151€ and 300€
   3. Between 301€ and 500€
   4. Between 501€ and 1000€
   5. Between 1001€ and 1500€
   6. Between 1501€ and 2000€
   7. Between 2001€ and 2500€
   8. Between 2501€ and 3000€
   9. Between 3001€ and 5000€
   10. Between 5001€ and 10.000€
   11. More than 10.000€
   12. Prefer not to say

**Stages of decision making**

People may be willing to do something for the environment, meaning consciously choosing to minimise or avoid the negative impact of their actions on the environment.

At this time, would you say you:

1. haven't begun to think about the choices;
2. haven't begun to think about the choices, but are interested in doing so;
3. are considering the options now;
4. are close to selecting an option;
5. have already made a decision, but are still willing to reconsider;
6. have already made a decision and are unlikely to change your mind.

**Decisional Conflict Scale**

People can decide to do something for the environment, in a range of behavioral options that go from “*considering the environment in everything they do*” to “*not considering it at all*”.

Considering the option you prefer, please evaluate the following statements.

*Answer options were: “Strongly Agree, Agree, Neither Agree nor Disagree, Disagree, Strongly Disagree”*

1. I know which options are available to me.
2. I know the advantages of each option.
3. I know the disadvantages of each option.
4. I am clear about which advantages matter most to me.
5. I am clear about which disadvantages matter most to me.
6. I am clear about which is more important to me (the advantages or the disadvantages).
7. I have enough support from others to make a choice.
8. I am choosing without pressure from others.
9. I have enough advice to make a choice.
10. I am clear about the best choice for me.
11. I feel sure about what to choose.
12. This decision is easy for me to make.
13. I feel I have made an informed choice.
14. My decision shows what is important to me.
15. I expect to stick with my decision.
16. I am satisfied with my decision.

# **Appendix K: Scale diagnosis results**

On the account of the correlation matrix, we dropped one item (i.e., 15. “I expect to stick with my decision”) because it did not show a correlation greater than |0.3|, thus indicating that this specific item seems to measure a different construct than decisional conflict. Calculation of Omega for the 15 items of the decisional conflict scale was .91. Based on the PCA, both a one-component solution and a three-components solution were possible for DC scale items. Specifically, the one-component solution was supported by the visual inspection of the scree plot, Figure K.1, [1] while the three-components solution was supported by the rotated factor loading and eigenvalue, Table K.1. Since previous literature showed mixed findings and multiple possibilities regarding the factor structure solution [2], and other authors [3] have used the one-component solution already, the one-component solution was considered the best and most parsimonious model for this study, and one-component PCA was run, Table K.2.

**Figure K.1**

*Scree plot of the factor analysis for the Decisional Conflict Scale with 15 items.*


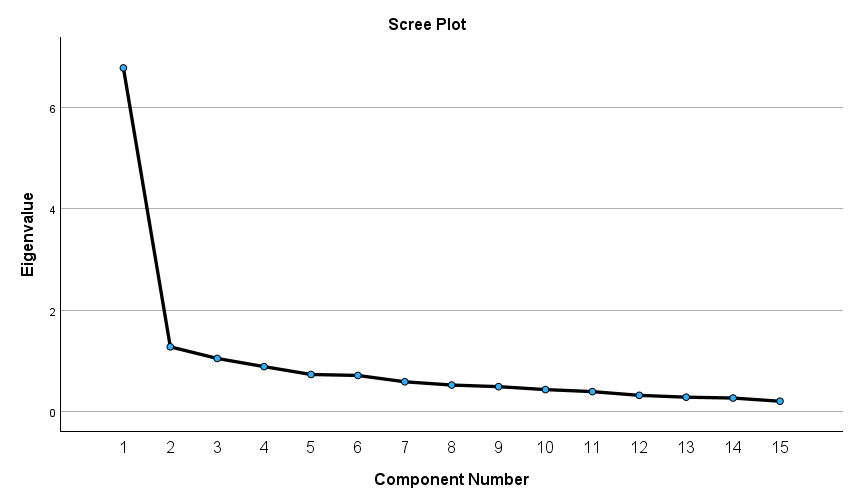


**Table K.1**

*Rotated Component Matrix for PCA with Varimax Rotation of a Three Component Scale (N=418).*

| DCS items | Rotated Component Coefficients | | |
| --- | --- | --- | --- |
|  | 1 | 2 | 3 |
| Factor 1: Decision | | | |
| 11. I feel sure about what to choose | **.77** | .25 | .20 |
| 16. I am satisfied with my decision | **.73** | .26 | .16 |
| 12. This decision is easy for me to make. | **.67** | .16 | .09 |
| 13. I feel I have made an informed choice. | **.65** | .36 | .32 |
| 8. I am choosing without pressure from others | **.60** | -.02 | .23 |
| 10. I am clear about the best choice for me. | **.57** | .48 | .31 |
| 14. My decision shows what is important to me. | **.57** | .50 | .04 |
| 9. I have enough advice to make a choice. | **.56** | .15 | .46 |
| 7. I have enough support from others to make a choice. | **.37** | .07 | .34 |
| Factor 2: Values Clarity | | | |
| 5. I am clear about what disadvantages matter most to me. | .10 | **.84** | .27 |
| 4. I am clear about what advantages matter most to me. | .16 | **.82** | .24 |
| 6. I am clear about which is more important to me (the advantages or the disadvantages) | .33 | **.70** | .20 |
| Factor 3: Informed | | | |
| 3. I know the disadvantages of each option. | .16 | .24 | **.85** |
| 2. I know the advantages of each option. | .22 | .26 | **.82** |
| 1. I know which option are available to me. | .33 | .29 | **.55** |

*Note.* Extraction Method: Principal Component Analysis. Rotation Method: Varimax with Kaiser Normalization. The overall Kaiser-Meyer-Olkin (KMO) measure was .91 with individual KMO measures all greater than .80. Bartlett's test of sphericity was statistically significant (*p* < .001), indicating that the data was likely factorizable.

**Table K.2**

*Principal Component Analysis of Decisional Conflict Scale (N=418).*

| Decisional Conflict Scale items | Component |
| --- | --- |
| 10. I am clear about the best choice for me. | .80 |
| 13. I feel I have made an informed choice. | .80 |
| 11. I feel sure about what to choose. | .75 |
| 16. I am satisfied with my decision. | .71 |
| 2. I know the advantages of each option. | .70 |
| 6. I am clear about which is more important to me (the advantages or the disadvantages) | .70 |
| 9. I have enough advice to make a choice. | .70 |
| 4. I am clear about which advantages matter most to me. | .68 |
| 14. My decision shows what is important to me. | .68 |
| 5. I am clear about which disadvantages matter most to me. | .66 |
| 3. I know the disadvantages of each option. | .66 |
| 1. I know which options are available to me. | .65 |
| 12. This decision is easy for me to make. | .58 |
| 8. I am choosing without pressure from others | .51 |
| 7. I have enough support from others to make a choice. | .46 |

*Note.* Extraction Method: Principal Component Analysis. The overall Kaiser-Meyer-Olkin (KMO) measure was 0.91 with individual KMO measures all greater than .80. Bartlett's test of sphericity was statistically significant (*p* < .001), indicating that the data was likely factorizable.

1. Cattell, R. B. (1966). The scree test for the number of factors. *Multivariate Behavioral Research, 1*(2), 245-276. <https://doi.org/10.1207/s15327906mbr0102_10>
2. Garvelink, M. M., Boland, L., Klein, K., Nguyen, D. V., Menear, M., Bekker, H. L., Eden, K. B., LeBlanc, A., O'Connor, A. M., Stacey, D., & Legare, F. (2019). Decisional Conflict Scale Use over 20 Years: The Anniversary Review. *Med Decis Making, 39*(4), 301-314. <https://doi.org/10.1177/0272989X19851345>
3. Gültzow, T., Smit, E. S., Crutzen, R., Jolani, S., Hoving, C., & Dirksen, C. D. (2022). Effects of an Explicit Value Clarification Method With Computer-Tailored Advice on the Effectiveness of a Web-Based Smoking Cessation Decision Aid: Findings From a Randomized Controlled Trial. *Journal of Medical Internet Research, 24*(7), e34246. <https://doi.org/10.2196/34246>

# **Appendix L: Language comparison**

### ***Italian Sample***

The logistic multiple regression model one was not statistically significant, χ^2^(4) = 3.36, *p* > .05. It became significant after stages were added in model two, χ^2^(5) = 21.94, *p* < .001. The full model explained 15.4% (Nagelkerke *R^2^*) of the variance in DC. Of the five predictor variables, only stages were statistically significant (B= -0.48, 95% CI 0.50 to 0.78, *p* < .001).

The hierarchical multiple regression model of gender, age, income and education to predict DC was not statistically significant, *R*^2^ = .04, *F*(4, 197) = 1.85, *p* > .05 [adjusted *R*^2^ = .02]. It became significant when stages were added, *R*^2^ = .14, *F*(5, 196) = 6.64, *p* < .001 [adjusted *R*^2^ = .12]. Only age was significant in model one (B= -1.59, 95% CI -3.11 to -0.07, *p* = .040), but after stages were added it became non-significant. Of the five predictor variables, only stages (B= -3.05, 95% CI -4.99 to -1.84, *p* < .001), were significant in model two.

### ***English Sample***

The logistic multiple regression model one was not statistically significant, χ^2^(4) = 6.87, *p* > .05. The same was for model two, χ^2^(5) = 10.26, *p* > .05.

In the hierarchical multiple regression, one participant was excluded because of a leverage value higher than 0.2. The model of gender, age, income and education to predict DC was not significant, *R*^2^ = .11, *F*(4, 67) = 1.97, *p* > .05 [adjusted *R*^2^ = .05]. It became significant when stages were added, *R*^2^ = .26, *F*(5, 66) = 4.62, *p* < .001 [adjusted *R*^2^ = .20]. Of the five predictor variables, only stages (B= -4.95, 95% CI -7.59 to -2.30, *p* < .001) were statistically significant in model two.

### ***Dutch sample***

The logistic multiple regression model one was not statistically significant, χ^2^(4) = 4.50, *p* > .05. It became significant after stages were added in model two, χ^2^(5) = 17.09, *p* < .001. The full model explained 26.3% (Nagelkerke *R^2^*) of the variance in DC. Of the five predictor variables, only stages were statistically significant (B= -0.80, 95% CI 0.27 to 0.75, *p* = .002).

In the hierarchical multiple regression, one participant was excluded because of a leverage value higher than 0.2, and one participant was excluded because of studentized deleted residual with a standard deviation greater than ± 3. The model of gender, age, income and education to predict DC was not significant, *R*^2^ = .12, *F*(4, 71) = 2.31, *p* > .05 [adjusted *R*^2^ = .12]. It became significant when stages of decision making were added, *R*^2^ = .37, *F*(5, 70) = 8.22, *p* < .001 [adjusted *R*^2^ = .33]. Age was significant in model one (B= -2.37, 95% CI -4.47 to -0.26, *p* = .028), but after stages were added it became non-significant. Of the five predictor variables, only stages (B= -5.40, 95% CI -7.43 to -3.38, *p* < .001) were significant in model two.

**Table L.1**

*Frequency table of participants sorted by language (N= 418).*

|  | Dutch  (n= 82) | English  (n = 86) | Italian  (n = 250) |
| --- | --- | --- | --- |
| **Gender, n (%)** | **82 (100.0)** | **85 (98.8)** | **248 (99.2)** |
| Men | 26 (31.7) | 24 (27.9) | 92 (36.8) |
| Women | 56 (68.3) | 59 (68.6) | 156 (62.4) |
| Non-binary | 0 | 2 (2.3) | 0 |
| Missing | 0 | 1 (1.2) | 2 (0.8) |
| **Age, n (%)** | **82 (100.0)** | **86 (100.0)** | **250 (100.0)** |
| 18-30 | 39 (47.6) | 76 (88.4) | 94 (37.6) |
| 31-40 | 12 (14.6) | 9 (10.5) | 26 (10.4) |
| 41-50 | 7 (8.5) | 0 | 54 (21.6) |
| 51-60 | 15 (18.3) | 1 (1.1) | 65 (26.0) |
| 61-70 | 6 (7.3) | 0 | 9 (3.6) |
| 71-80 | 3 (3.7) | 0 | 2 (0.8) |
| >80 | 0 | 0 | 0 |
| Missing | 0 | 0 | 0 |
| **Income, n (%)** | **78 (90.7)** | **74 (86.0)** | **207 (82.8)** |
| < 150€ | 7 (8.5) | 5 (5.8) | 10 (4.0) |
| 151€ – 300€ | 6 (7.3) | 4 (4.7) | 5 (2.0) |
| 301€ – 500€ | 4 (4.9) | 7 (8.1) | 4 (1.6) |
| 501€ – 1000€ | 10 (12.2) | 25 (29.0) | 11 (4.4) |
| 1001€ – 1500€ | 4 (4.9) | 9 (10.5) | 45 (18.0) |
| 1501€ – 2000€ | 6 (7.3) | 6 (7.0) | 46 (18.4) |
| 2001€ – 2500€ | 12 (14.6) | 1 (1.2) | 24 (9.6) |
| 2501€ – 3000€ | 6 (7.3) | 4 (4.7) | 20 (8.0) |
| 3001€ – 5000€ | 15 (18.3) | 4 (4.7) | 29 (11.6) |
| 5001€ – 10.000€ | 8 (9.7) | 8 (9.3) | 6 (2.4) |
| >10.000€ | 0 | 1 (1.2) | 7 (2.8) |
| 1501€ – 2000€ | 6 (7.3) | 6 (7.0) | 46 (18.4) |
| Missing | 4 (4.9) | 12 (14.0) | 43 (17.2) |
| **Education level, n (%)** | **82 (100.0)** | **86 (100.0)** | **246 (98.4)** |
| Low | 1 (1.2) | 0 | 12 (4.8) |
| Medium | 21 (25.6) | 38 (44.2) | 100 (40.0) |
| High | 60 (73.2) | 48 (55.8) | 134 (53.6) |
| Missing | 0 | 0 | 4 (1.6) |

1. The dataset provided on Open Science Framework (<https://osf.io/mgvjb/>) has already been recoded and filtered for the inclusion criteria. Instead, the syntax file is comprehensive of all the recoding and cleaning steps. [↑](#footnote-ref-1)
